# The Usefulness of Exosomes in Accelerating Healing and Preventing Complications in Behçet’s Disease: A Case Report

**DOI:** 10.7759/cureus.74476

**Published:** 2024-11-26

**Authors:** Marwa H Elajami

**Affiliations:** 1 Gynecology, CosmeSurge Group, Dubai, ARE

**Keywords:** bartholin’s cyst, behçet’s disease, exosomes, labiaplasty, wound healing

## Abstract

Behçet’s disease (BD) is a systemic auto-inflammatory vasculitis. The clinical pictures of BD involve the skin and mucosal membranes such as oral and genital ulcers, ocular lesions, cardiovascular, gastrointestinal, muscular, nervous systems, and joints. A 38-year-old woman was repeatedly suffering from oral, genital, and ocular lesions, wound dehiscence after any surgical procedure, and joint pain. She has a family history where multiple relatives were affected by BD. The patient had an abdominoplasty two years ago with delayed wound healing and keloid formation. The patient presented with an asymmetrical labia majora with multiple lumps after fat transfer, multiple vaginal cysts, and a chronic Bartholin’s cyst for three years. She consulted surgeons to correct the deformity and for cyst excision, but there was a great risk of keloid formation, dehiscence, and infections. This was a challenge for her surgery. The patient was on colchicine and corticosteroids. BD affects multiple systems, most commonly the healing of the skin and the mucous membranes. This causes difficulties in deciding to undergo surgery because of the risks. The diversity of the manifestations of BD requires a spectrum of pre-surgical adjustments in inflammatory markers, medication doses, and improving general patient conditions. Furthermore, the intra-operative and post-operative use of the recent regenerative medicine methods might provide the best surgical healing outcomes in these groups of patients. This is most challenging with BD but can be achieved regardless.

## Introduction

Behçet’s disease (BD) is a systemic disorder characterized by chronic inflammation of blood vessels, leading to multisystemic involvement. The disease often manifests with oral and genital ulcers, skin lesions, and ocular inflammation, but its impact on wound healing is particularly significant. Persistent inflammation can impair the healing process, causing complications such as wound dehiscence, keloid formation, and infections, which are critical considerations for surgical patients [1].

It is a chronic, relapsing systemic disorder characterized by variable clinical manifestations, including oral and genital aphthae, cutaneous lesions, ocular, gastrointestinal, neurologic involvement, and arthritis [1,2].

BD is more common in countries located along the “Silk Road,” an ancient trading route connecting Asia with Middle and Southern Europe - about 14-20 per 100,000 people - and the highest incidence is noted in Turkey (20-421/100,000) [1,3]. Given its prevalence along the Silk Road, BD represents a significant health burden in regions with limited access to advanced therapies. Innovative approaches like exosome therapy could provide accessible and effective options for managing complications associated with BD.

The diagnosis is based on clinical scores. The most used criteria sets are the revised International Criteria for Behçet’s Disease (ICBD), updated in 2014 [4].

Regenerative medicine deals with restoring the normal function of human cells and tissues. Stem cells, platelet rich plasma, and exosomes are examples of regenerative medicine. We chose to apply exosomes derived from rose stem cells in patient's surgery because it is a cell-free and hypoimmunogenic treatment that promotes regeneration, cellular repair, and collagen synthesis without depending on the patient’s platelet's function, which may be affected by colchicine and steroids. In addition, exosomes have anti-inflammatory properties.

Exosomes are tiny vesicles naturally (diameter of approximately 30-200 nanometers) produced by cells that play a critical role in cell communication. They carry bioactive molecules like RNA and proteins that can promote tissue repair, reduce inflammation, and support collagen synthesis [5]. Unlike stem cell therapies, exosomes are cell-free, making them safer and less immunogenic, especially for patients with compromised immune systems or those taking medications such as steroids [5]. Engineering exosomes are promising materials for the next generation of nanomedicine for therapy with non-cytotoxic effects and low immunogenic profiles [5].

The most recognized mechanism for formation of exosome is driven by the endosomal sorting complexes required for transport (ESCRT) complexes [5]. Once released, exosomes fuse with the plasma membranes of recipient cells to deliver their contents [6].

Being vasculitis, the involvement of skin and mucous membranes by the disease has different manifestations, such as wound dehiscence, wound infections, and keloid formation. These are frequent challenges BD affected patients face during wound healing, more commonly when inflammatory markers are elevated pre-operatively. In the context of BD, where impaired wound healing is a significant concern, exosomes represent a promising therapeutic option. Their ability to modulate inflammation and enhance tissue regeneration could address the common complications faced by patients undergoing surgery.

However, the use of recent regenerative medicine might hope to be successful in improving healing as well as the cosmetic shape after surgeries. This is especially likely when blended with maximum adjustment of the pre-operative inflammatory parameters and the doses of pre-operative anti-inflammatory drugs. The clinical case presented illustrates the significance of the integration of regenerative medicines combined with surgical skills. This can help us decrease complications while healing for high-risk cases like BD.

## Case presentation

Ethical approval and written informed consent were obtained from the NMC research center and patients, respectively. The patient was 38 years old, para 2, with a known case of BD discovered in 2021 on corticosteroid (that was prescribed by her rheumatologist; then stopped by the patient herself in the same year). The patient took Colchicine 0.5mg daily and had an abdominoplasty with fat transfer to the labia majora in 2022 which ended up with delayed healing. Her wound took two months to heal, the scar developed a pigmented raised keloid, fat cysts were found in the labia majora, as well as obvious asymmetry in the labia majora (Table 1).

**Table 1:** International criteria for BD point score system Behçet’s diagnosis is considered if the patient presents ≥ 4 points. *Pathergy test is optional and the primary scoring system does not include pathergy testing. However, where pathergy testing is conducted one extra point may be assigned for a positive result. Adopted from Davatchi et al., The International Criteria for Behcet’s Disease (ICBD) [4]. Copyright permission to use Table 5 from [4] has been obtained (license number 5906320792034) from John Wiley and Sons dated November 12, 2024.

International criteria for Behcet’s Disease	Points
Genital ulcer	2
Oral aphthosis	2
Ocular lesion	2
Vascular manifestations	1
Skin lesion	1
Neurological manifestations	1
Vascular manifestations	1
Positive pathergy test (optional)*	1

In addition, she was suffering from chronic Bartholin’s cysts and vaginal cysts that were managed by needle aspiration 10 months before the patient presented to my clinic. The Bartholin’s cyst returned, and the patient was suffering from dyspareunia and pain at the site of the vulvo-vaginal cysts. She had multiple liposuctions under local anesthesia by her plastic surgeon to remove the fat lumps and to improve the equality of the labia majora, but unfortunately, her problems were not resolved. She presented to my cosmetic gynecology clinic with the aforementioned complaints and was feeling hopeless, thinking that she can no longer improve the quality of her sexual life and the cosmetic shape of her vulva.

On vaginal examination, Bartholin’s cyst was nontender 2x2 cm, there were two vaginal cysts at the midline of the lower third of the vagina, scarred and deficient perineum, and both labia majora showed obvious asymmetry while standing and laying on her back (right labia majora is 2 cm larger and wider than the left side; both sides are hypertrophied and huge in size). Her abdomen showed a pigmented raised keloid due to a transverse abdominoplasty. The keloid was about 30 cm in length.

The patient's pre-operative laboratory and radiologic results were reviewed. Serum electrolytes, liver, and renal functions were within normal limits. The patient initially had an elevated C-reactive protein (CRP) level of 4.2 mg/L, which decreased to 1.2 mg/L after doubling the Colchicine dose. Similarly, the erythrocyte sedimentation rate (ESR) decreased from 41 mm/hr to 26 mm/hr following the same adjustment. Hemoglobin was measured at 12.1 g/dL, with a platelet count of 283,000, and a normal coagulation profile.

**Figure 1:**
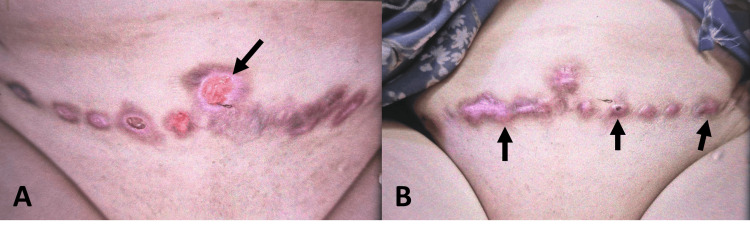
Wound healing complications after an abdominoplasty in the same patient with Behçet’s disease (no use of regenerative medicine) (A, B) Pigmented keloid (black arrows) with ulceration of the abdominoplasty wound and inflammatory wound attacks after surgery in Behcet’s disease patients. The long duration of wound healing takes months in those patients with bad cosmetic appearance.

**Figure 2:**
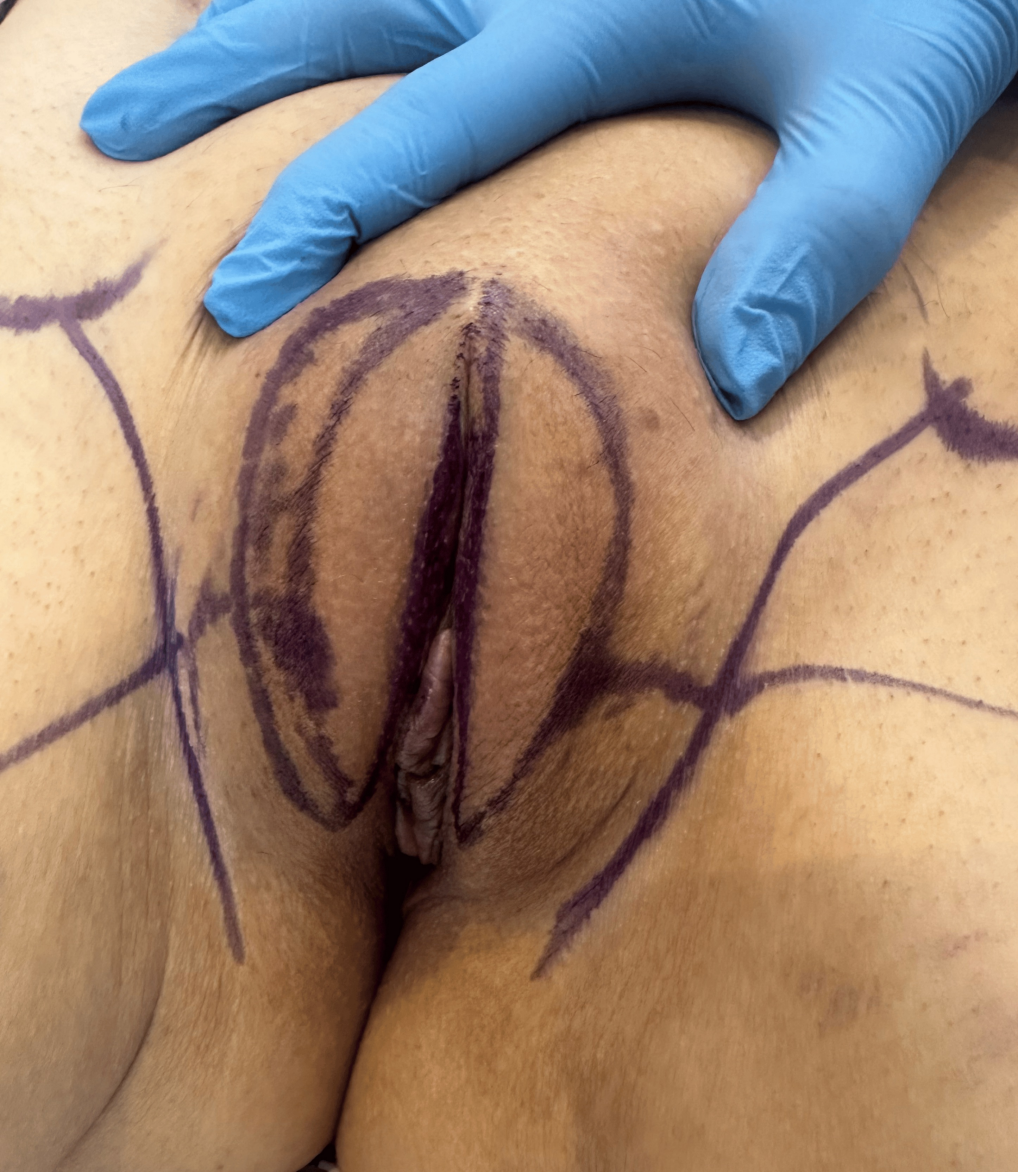
Pre-operative markings. Precise marking to reduce the asymmetry and the wound size are essential to avoid revision surgeries in high-risk cases like Behcet’s disease.

**Figure 3:**
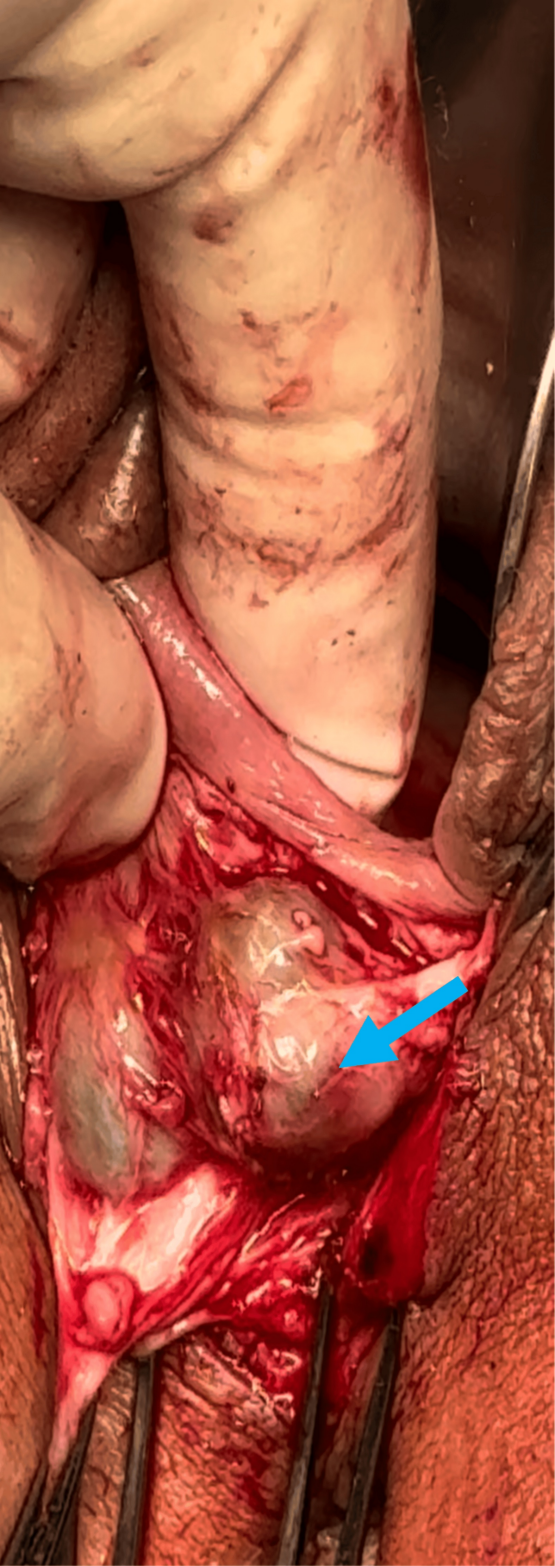
Intra-operative picture. The picture demonstrates the vaginal cysts found communicating with the recurrent Bartholin’s cyst in the midline (blue arrow), causing dyspareunia and interfering with the sexual life of the patient. Excision is done to avoid the recurrence.

The planned surgical procedure, discussed thoroughly with the patient, included excision of Bartholin’s gland and vaginal cysts, reduction of the labia majora, and a perineoplasty. Prior to surgery, the strategy was to optimize the patient's inflammatory markers, in consultation with a rheumatologist, to address any potential drug interactions and obtain further recommendations. Intraoperatively, I aimed to minimize tissue trauma by utilizing efficient surgical techniques and reducing the size of the skin wound through careful mapping. Figures 1A, 1B show the wound healing complications after abdominoplasty. Figure 2 shows the pre-operative markings applied to the patient. Figure 3 describes the vaginal cysts communicating with the Bartholin’s cyst.

**Figure 4:**
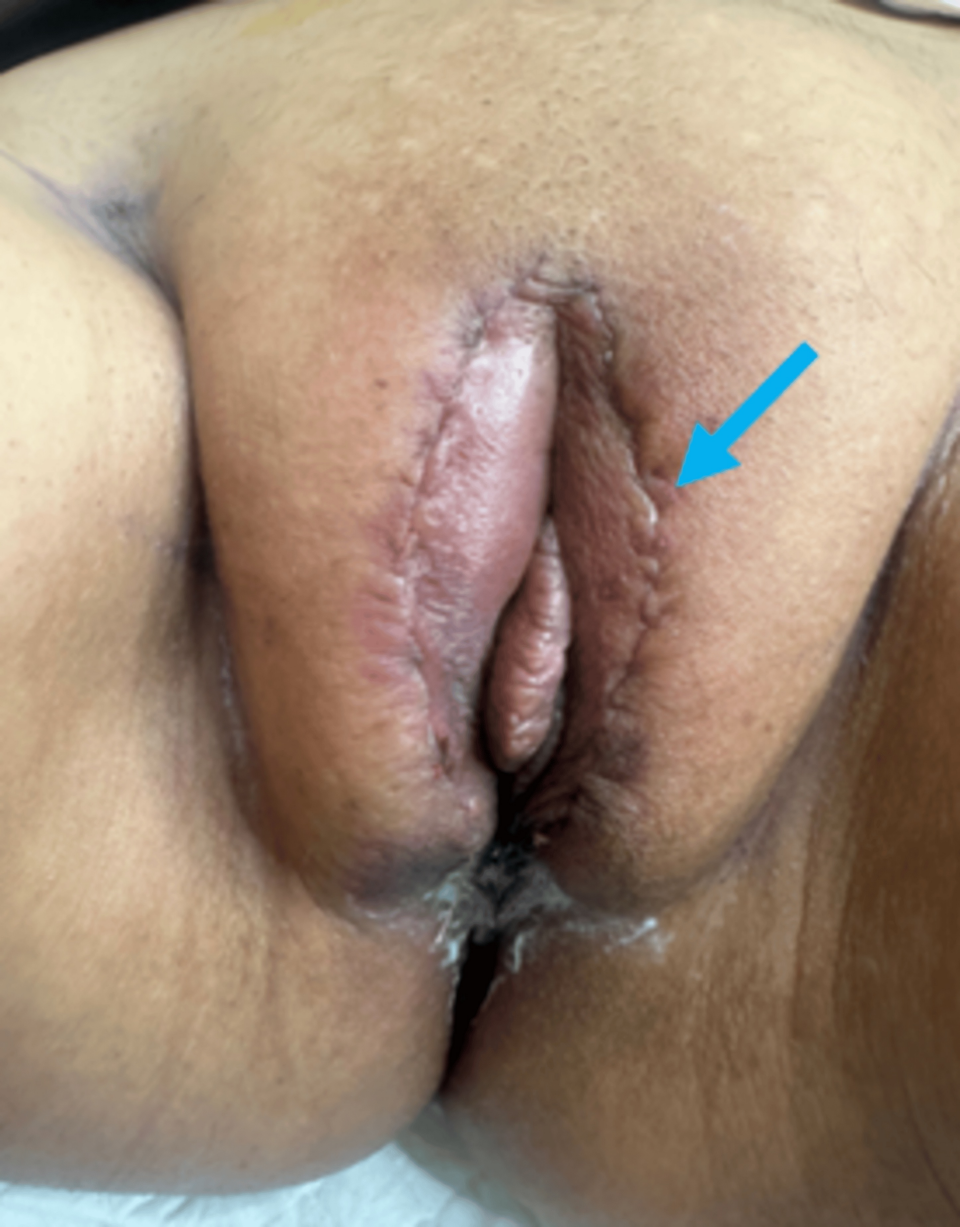
Post-operative seven days. The image showing initial promising wound healing improvement, swelling, and bruises are expected. No wound gapping was the main promising healing sign. Exosomes applied in two layers protocol.

**Figure 5:**
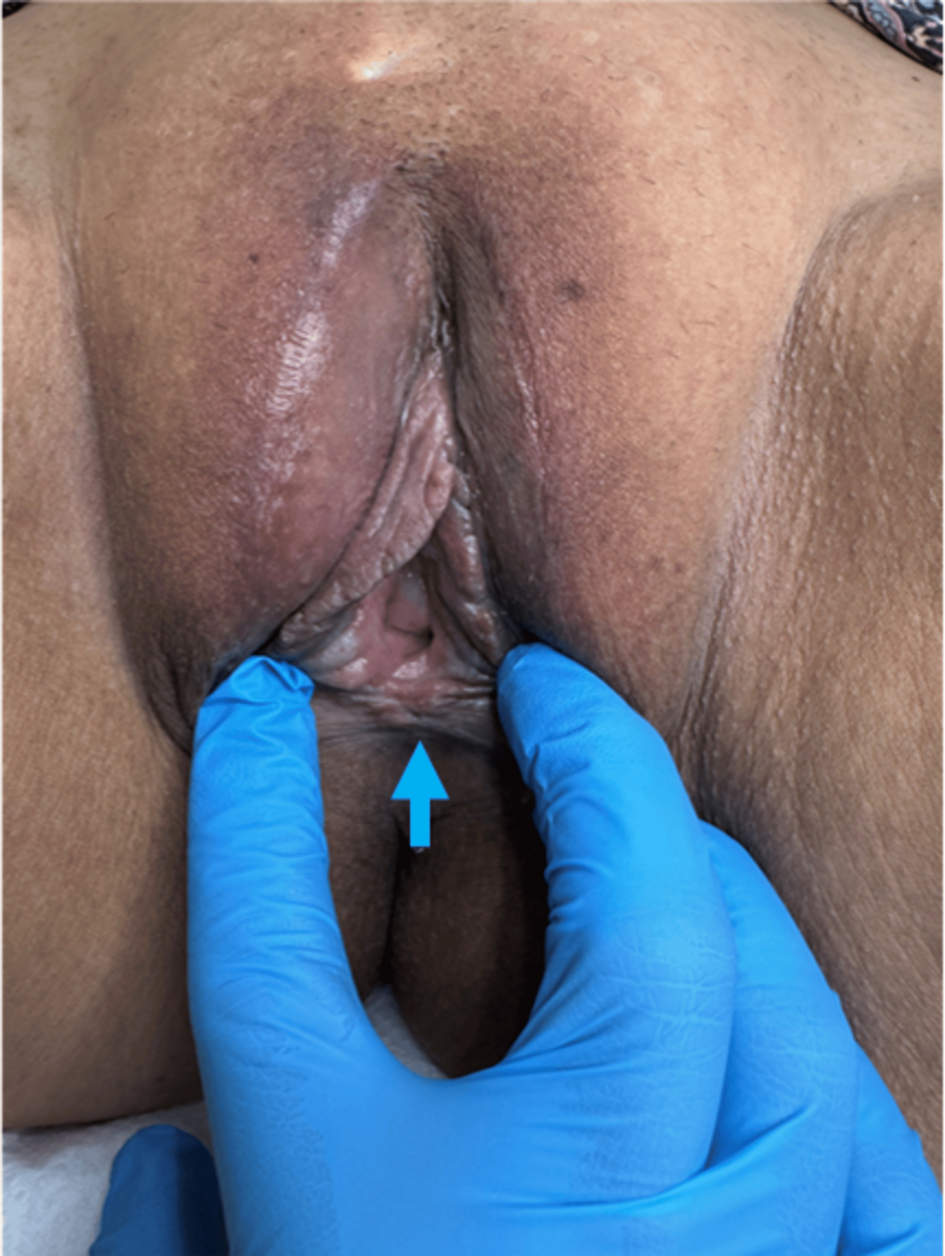
Four weeks post-operative. Image showing the complete healing of the vaginal and labia majora wounds and exosomes re-applied topically in two layers protocol to enhance the absorption of maximum number of exosomes.

**Figure 6:**
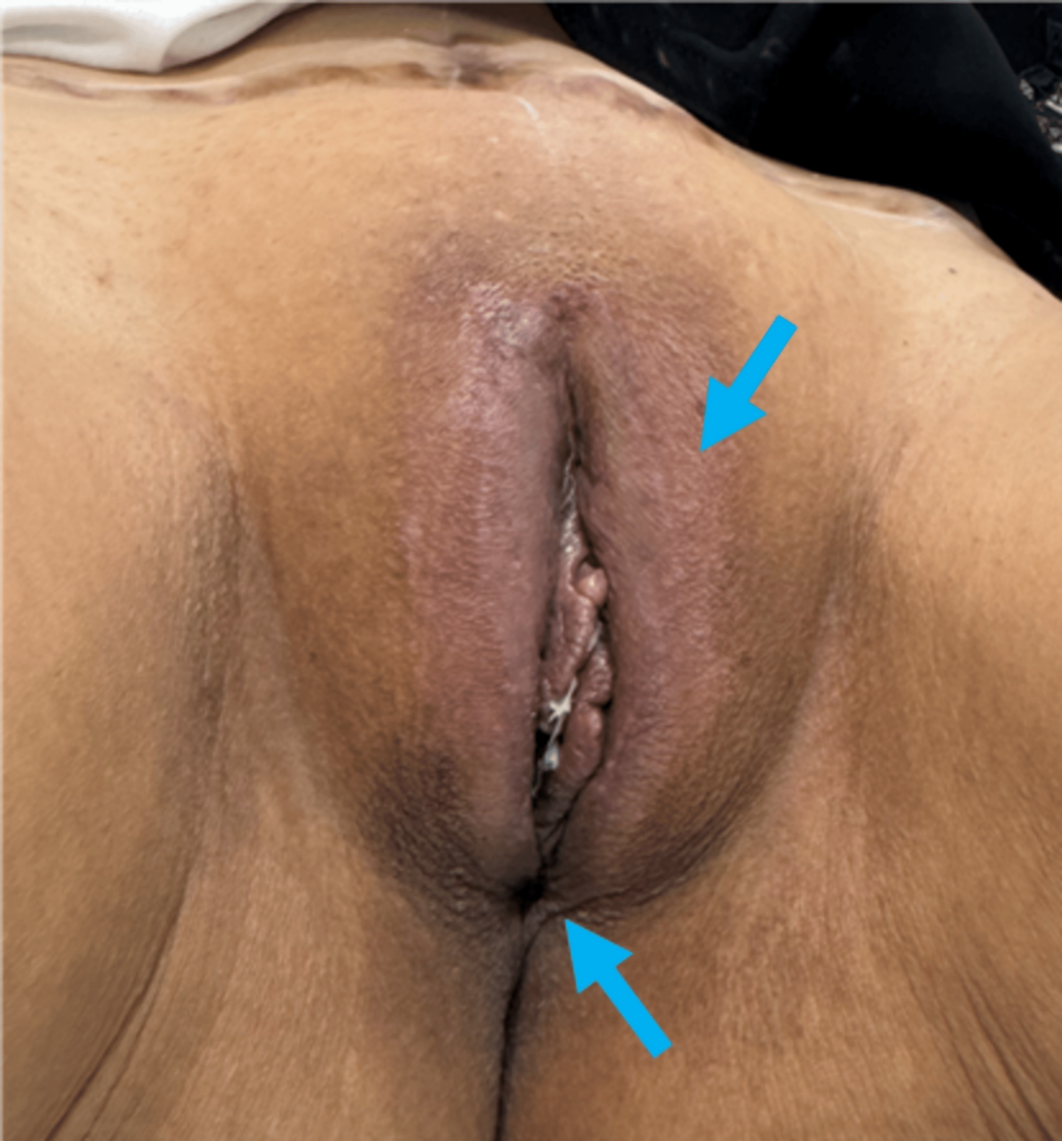
Result after six weeks post-operative. The final result at six weeks post-operative demonstrated the symmetrical size after the reduction of labia majors, wound healing is complete, no wound complications and the patient returned to her normal sexual life and is satisfied with the healing journey and the cosmetic results (the perineal and labia scars almost undetectable; blue arrow).

Post-operatively, the Colchicine dose would be doubled from 0.5 mg/day to 1.0 mg/day and maintained for one month following surgery. Additionally, I planned to apply exosomes topically during the operation and subsequently at one week and four weeks post-operatively. The exosome application protocol involved applying two layers of exosomes topically, with each layer spaced 10 minutes apart. Figures 4-6 show the patient's post-operative improvement.

## Discussion

BD is a complex widespread inflammatory disorder that affects both large and small blood vessels [5,6]. It presents major challenges across a wide range of clinical situations, especially in the perioperative care of surgical patients. The inflammation of blood vessels associated with BD hinders healing processes, thereby increasing the risks that accompany surgical procedures. These risks include a heightened vulnerability to wound dehiscence, keloid formation, and infections [7]. This case emphasizes the essential need to recognize these risks and emphasizes the necessity for innovative approaches to improve the healing process.

In this instance, the patient had a history of delayed wound healing after undergoing an abdominoplasty and presented with notable vulvar asymmetry and recurrent Bartholin's cysts. These manifestations of BD, particularly considering impaired healing, required careful preoperative planning, intraoperative techniques, and postoperative management. Importantly, despite treatment with corticosteroids and colchicine, the patient's inflammatory markers, specifically C-reactive protein (CRP) and erythrocyte sedimentation rate (ESR), remained enhanced, necessitating adjustments in medication dosage and an emphasis on optimizing her widespread inflammatory status prior to surgery.

Recent developments in regenerative medicine, including the application of exosomes, offer a promising supplementary treatment option for this patient. Exosomes (extracellular vesicles with about 30-150 nm diameter) [8] are capable of assisting cellular communication and delivering bioactive molecules such as messenger RNA (mRNA), microRNA (miRNA), and peptides, thereby providing a novel method to enhance tissue repair and modulate inflammation without the immunogenic concerns associated with other cell-based therapies [9-11].

In this case, exosomes derived from rose stem cells were selected due to their anti-inflammatory characteristics and their capacity to promote collagen synthesis and tissue regeneration. This cell-free approach avoids the potential risks linked to stem cell therapies, which may depend on the patient's compromised platelet function, particularly in those taking medications such as colchicine and corticosteroids [9,12].

The intraoperative use of exosomes, with careful surgical techniques to minimize tissue trauma, was intended to prevent the recurrence of complications that the patient had previously experienced, including wound dehiscence and keloid formation [13]. The patient’s positive postoperative experience, marked by the absence of infection or wound complications, indicates that the combined strategy of managing preoperative inflammation and using regenerative medicine may present an effective approach for high-risk patients with BD.

## Conclusions

BD presents considerable challenges in surgical management, primarily due to its effect on wound healing and inflammation. In this instance, the incorporation of regenerative medicine, particularly the utilization of exosomes, was critical in enhancing the healing process and averting complications. Utilizing exosomes, with the control of preoperative inflammatory markers and surgical precision, led to successful surgical outcomes without important complications. However, the effect of other treatments given to the patient cannot be ignored. This case emphasizes the potential of exosome therapy to become a valuable resource in the management of high-risk surgical patients with BD. Further investigation and extensive clinical studies are necessary to ascertain the effectiveness and safety of exosome therapy in managing BD and other inflammatory conditions that impact wound healing.
